# On the Impact of the Fabrication Method on the Performance of 3D Printed Mixers

**DOI:** 10.3390/mi10050298

**Published:** 2019-04-30

**Authors:** Mojtaba Zeraatkar, Daniel Filippini, Gianluca Percoco

**Affiliations:** 1 Department of Mechanics, Mathematics and Management, Polytechnic University of Bari, 70126 Bari, Italy; mojtabazeraatkar@poliba.it; 2 Optical Devices Lab, Department of Physics, Chemistry and Biology, Linköping University, 58183 Linköping, Sweden; daniel.filippini@liu.se

**Keywords:** micromixing, fused deposition modeling, stereolithography, polyjet

## Abstract

A wide variety of 3D printing technologies have been used for the fabrication of lab-on-a-chip (LOC) devices in recent years. Despite the large number of studies having examined the use of 3D printing technologies in microfluidic devices, the effect of the fabrication method on their performance has received little attention. In this paper, a comparison is shown between unibody-LOC micro-mixers, a particular type of monolithic design for 3D printed LOCs, fabricated in polyjet, stereolithography (SLA) and fused deposition modelling (FDM or FFF) platforms, paying particular attention to the inherent limitations of each fabrication platform and how these affect the performance of the manufactured devices.

## 1. Introduction

Lab-on-a-chip (LOC) [1] devices have a wide variety of chemical and biological applications including biomedical diagnostics, DNA and protein analysis, food safety control, drug development. LOC devices have several advantages compared with wet chemistry procedures, including compact size, automatic operation, fast and highly sensitive detection and reduced reagents consumption [2]. Classical LOC systems are typically designed and constructed in laboratories, with processes that demand specialized operators and costly processes as well as dedicated infrastructure such as clean rooms. The advent of modern additive manufacturing techniques has allowed for most of the fabrication skills to be transferred to 3D printers, thereby simplifying the development process and reducing the required infrastructure to a single platform. The versatility offered by 3D printers not only shortens the optimization process but numerous modifications to be made at each iteration cycle, something that would be impossible in a traditional manufacturing process. In addition, 3D printing offers the ability to create truly three-dimensional architectures that are ideal for microfluidic purposes [3,4].

In particular, the unibody-LOC concept simplifies fabrication, assembly and connectivity as the devices are designed as a single monolithic structure with connectors already embedded. A wide variety of 3D-printing technologies have been used for the fabrication of microfluidic devices. Among the most practical are fused deposition modelling (FDM or FFF), stereolithography (SLA), polyjet, two-photon lithography, selective laser sintering, and layered hydrospinning [5]. 3D printed unibody-LOC was originally developed for SLA printers [6,7,8] and features open channels, at least on one side, to enable easy removal of uncured resin with minimal exposure to solvents. This allows for the fabrication of geometrically consistent channels of arbitrary length. Closing these channels relies on adhesive tape or thin films to produce an effective seal, which is made possible through the good surface finish in SLA printouts. FDM was firstly used in 2001 as a method to fabricate templates for soft lithography of microfluidic devices [9]. In 2012, FDM was demonstrated for the direct fabrication of microfluidic chemical reaction-ware [10]. Over 100 articles related to 3D printing and microfluidic devices discuss the different printing methods and devices that have been made with each method [5,11,12,13,14]. With a wide range of 3D printers, the suitability for microfluiadic devices has been studied by comparing different SLA printers [15], different polyjet printers [16], and comparisons between polyjet and FDM approaches [17]. 

Despite the ubiquity of studies on the use of 3D printing technologies in microfluidic devices, cross-platform comparisons with regard to microfluidic performance are less common. MacDonald et al. [18] compared three different printing methods (FDM, polyjet, and SLA) used for the fabrication of a simple Y-shaped micro-mixer with regard to fabrication method and mixing performance. Micro-mixers are crucial components in microfluidic devices as they facilitate sample dilution, reagent homogenization, and chemical or biological reactions [19]. The flow in microfluidic devices is often laminar (characterized by low Reynolds numbers resulting from small channel sizes), which makes it difficult to achieve effective mixing. As a result, mixing in microfluidic devices relies on diffusion between the different flows, which is inherently slower and requires long channels to attain a mixture. Over the past decades, various systems to enhance mixing efficiency in microfluidic devices have been introduced, which can generally be divided into two groups: passive and active [2,20,21]. Their main goal is to transform the laminar into turbulent flow by altering the structure or configuration of fluid channels (passive) [22] or using external energy sources (active) [23]. Mixing is generally induced by two phenomena: diffusion [24] and chaotic advection [25].

Due to continual progress in other 3D-printing techniques, fine features that used to be exclusive to SLA can now be obtained also with FDM and polyjet platforms [26], which bring the benefit of precisely defined printing materials. In the present paper, unibody-LOC micro-mixers made through Polyjet, SLA, and FDM platforms are examined and compared in terms of performance and limitations of the different fabrication platforms. Performance will be assessed in terms of completeness of mixing, channel length, and related fabrication process. 

## 2. Materials and Methods

### 2.1. Fabrication of Unibody Micro-Mixers

**Figure 1:**
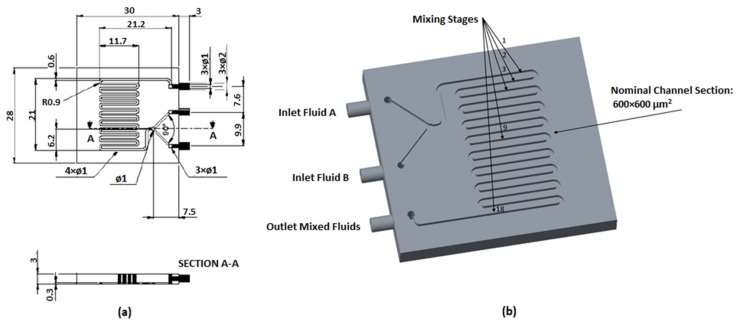
(**a**) Computer-assisted design (CAD) drawing of the designed micro-mixer with dimensions in mm; (**b**) 3D model of unibody passive micro-mixer.

Classical Y-shaped connected channels were fabricated with a serpentine to allow the observation of the mixing process using the unibody-LOC architecture (Figure 1). The main elements of such devices are: (i) inlets: the input connectors. The device was equipped with two inlets to allow two different flows to join in the serpentine; (ii) main channel: a serpentine-like channel, 600 µm wide and 600 µm high, consisting of 18 turns; (iii) outlet: the output connector, of the same size as the inlets.

Three different processes were exploited to fabricate unibody-LOCs: (i) polyjet (Stratasys Objet 30, Masmec spa, Biomed Division, Bari, Italy); (ii) SLA (Formlabs Form 2, Solfa IT Inc., Altamura, Italy); (iii) FDM or FFF (Ultimaker 3, Polytechnic University of Bari, Bari, Italy) (see Table 1 for a summary). In this paper several tests were described, conducted on three identical devices fabricated with polyjet, SLA and FDM. Details and parameters of process are reported in Table 1.

Printed devices were cleaned and tape (3M Ruban Adhesif Scotch^®^, St. Paul, MI, USA) was used to seal the surfaces, thus closing the open sides of the unibody channels.

### 2.2. Experimental Setup

Prior to image acquisition, devices were cleaned with water and dried using warm air. Compressed air jets were used to eliminate any drops of water trapped inside the channel.

**Figure 2:**
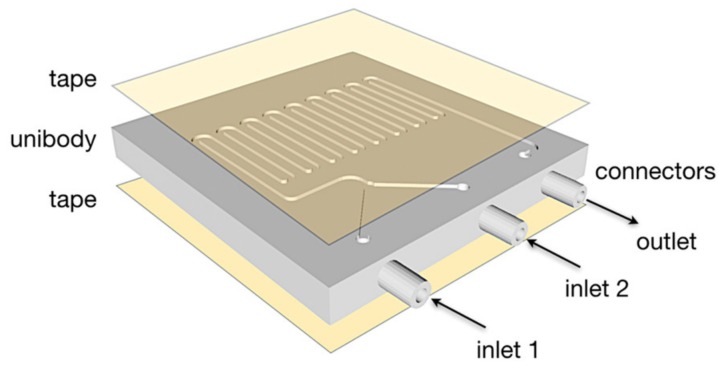
Transparent double-sided tape was applied to the top and bottom of the device.

Although the surface finish delivered by the 3D printing is sufficient for the solutions, the top and bottom surfaces were smoothed using abrasive finishing paper to ensure an effective seal (Figure 2).

**Figure 3:**
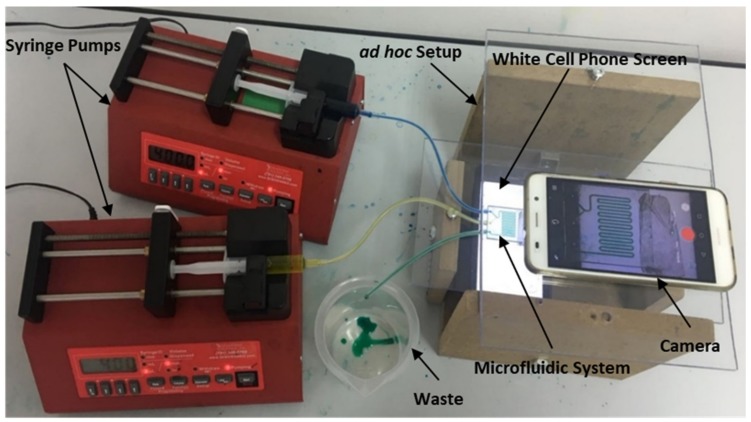
Experimental setup and detection system for testing our microfluidic systems.

Two different water solutions stained with yellow and blue food colour, at designated flow rates, were injected using two syringe pumps (Braintree, MA, USA, Mod. BS-300) actuating on 5 mL disposable syringes. An ad hoc setup was configured to characterize the devices. A bottom plate and double-sided adhesive tape were used to fix the micro-mixer devices in place. The camera was placed on the top plate for detection. A white cell phone screen was positioned below the lower plate to provide homogenous back-illumination (Figure 3). 

The unibody design which mechanically fits to the inner diameter of the coupling silicone pipe ensures a leakage-proof connection between the syringes and mixers. Four different flow rates through the main channel were tested: 10 µL/min, 50 µL/min, 200 µL/min, and 400 µL/min.

## 3. Results and Discussion 

**Figure 4:**
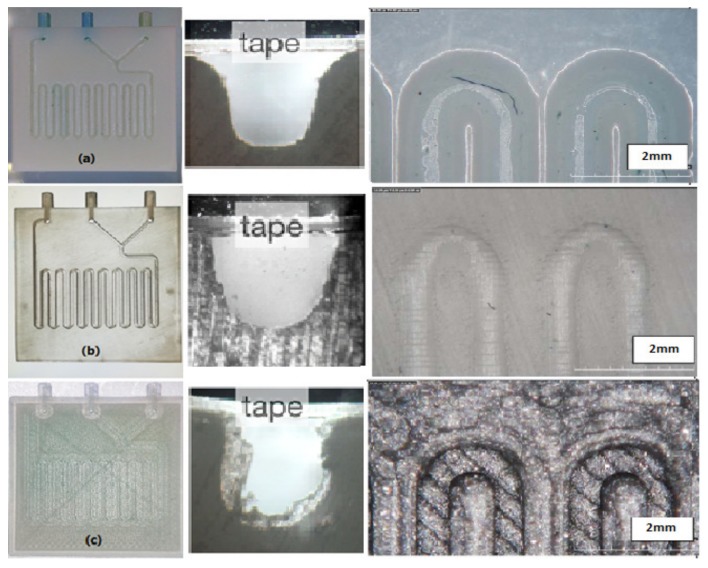
Fabricated micro-mixers by three different methods of printing. (**a**) micro-mixer with polyjet in cross-section and top view (**b**) micro-mixer with SLA in cross-section and top view (**c**) micro-mixer with FDM in cross-section and top view.

Figure 4 shows the fabricated micro-mixers with the three different printing methods; as regards 3D dimensional comparisons, please refer to [27]. The limited availability of printable materials and the high cost of the process are the main drawbacks of the polyjet process. The surface of the SLA micro-mixer is smoother (Figure 4b) compared to FDM, but the SLA method requires proprietary resins [28], whereas FDM employs common and well described polymers. The SLA device is more transparent, which facilitates observation and interpretation of the fluidic phenomena. The main advantages of FDM are its relatively low cost and the possibility of using different materials. The FDM LOC was also imaged with back illumination, whereas the polyjet was opaque and was imaged under ambient light. The 3D models of micro-mixers realized through the three fabrication methods present only minor differences between the measured and nominal values of the average channel depth (<20 µm). The cross sections in Figure 4 show the fidelity of the 3D printing methods to produce a square cross section. The polyjet method shows a higher fidelity than the SLA and FDM approaches. In particular, in FDM, due to the spreading of the polymer as it is extruded, the channel can be, in some sections, consistently smaller than the CAD model.

**Figure 5:**
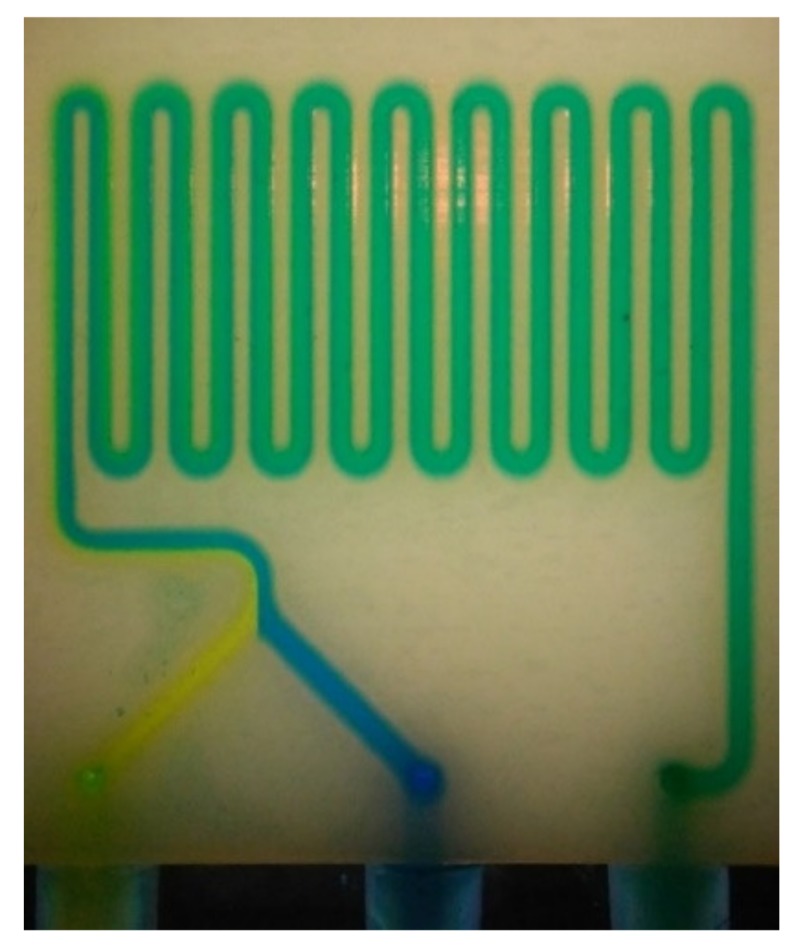
Complete mixing obtained with the polyjet micro-mixer at a flow rate of 10 µL/min.

**Figure 6:**
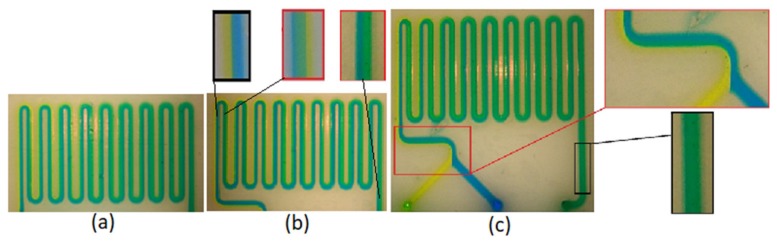
Mixing obtained in polyjet micro-mixer with different flow rates. (**a**) flow rate of 50 µL/min; (**b**) flow rate of 200 µL/min; (**c**) flow rate of 400 µL/min.

All fabricated micro-mixers showed comparable mixing efficiencies. In this analysis mixing completion and length of the required channel for complete mixing were investigated. For a flow rate of 10 µL/min in the polyjet mixer, the two flows running through the first section of the LOC form a clear boundary, which dissipates after few turns in the serpentine, leading to a homogeneous green colour (Figure 5). Several flow rates were tested to analyse the influence of the flow rate on length of the required channel and as a result of mixing time (Figure 6). When the flow rate is higher (50 µL/min) the boundary remains, since the channels length is not enough to allow the diffusion process (Figure 6a). For a flow rate of 200 µL/min, the flow is still laminar and simply stays a shorter time in the device to allow diffusional mixing (Figure 6b); however, at 400 µL/min the fluid becomes chromatically homogeneous at the end of the channel (Figure 6c), which indicates the onset of turbulence contribution (Figure 6c).

**Figure 7:**
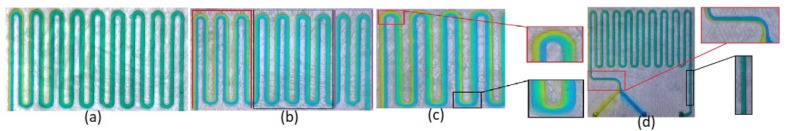
Mixing obtained in SLA micro-mixer. (**a**) flow rate of 10 µL/min; (**b**) flow rate of 50 µL/min; (**c**) flow rate of 200 µL/min; (**d**) flow rate of 400 µL/min.

**Figure 8:**
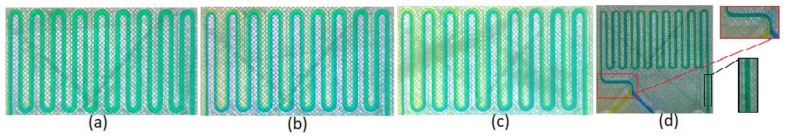
Mixing obtained in FDM micro-mixer. (**a**) flow rate of 10 µL/min; (**b**) flow rate of 50 µL/min; (**c**) flow rate of 200 µL/min; (**d**) flow rate of 400 µL/min.

Performing the same tests on the SLA and FDM mixers (Figure 7 and Figure 8) yielded complete mixing for flow rates of 10 μL/min and 400 μL/min, similar to the results for the polyjet mixer. At flow rates of 50 μL/min and 200 μL/min, mixing was incomplete and with a clearly visible boundary between the two fluids remaining. However, as the two fluids approach the outlet, this boundary becomes less distinguishable (Figure 7b,c and Figure 8b,c).

**Figure 9:**
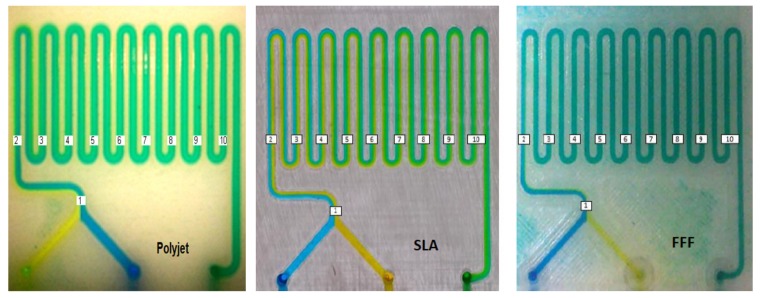
Regions of interest in the different microchannels.

**Figure 10:**
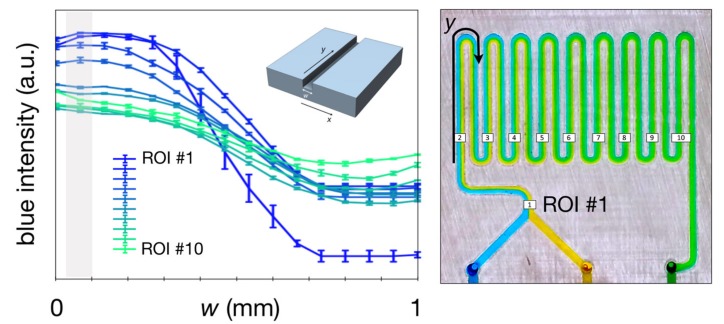
Diagram of the mixing trend in the microchannel using a blue colour scale. X-axis and Y-axis are considered in width and length of channel respectively.

These results show that by increasing the flow rate, the required channel length to achieve complete mixing needs to be increased as well. These results are consistent with theory [18], and in order to quantify and compare the mixing performance of these devices acquired images in a steady regime were analysed. Ten regions of interest (ROIs) were selected along the serpentine (Figure 9), and the spatial distribution of the colour intensity was recorded using a bespoke Matlab script. For each ROI covering the width of the channel (w, Figure 10) the pixels are averaged and the standard deviation calculated for every position along *w*. Figure 10 describes the intensity of the blue channel of the colour image, which illustrates the homogenization of the colour intensity along the serpentine, when the blue flow diffuses into the yellow flow becoming green.

**Figure 11:**
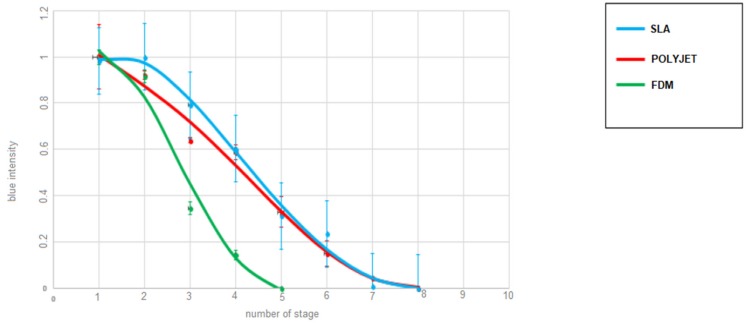
Required channel length for complete mixing (at a flow rate of 10 μL/min) for the different 3D printing methods.

In order to find the channel length that is necessary to achieve complete mixing, the blue intensity decay at the channels’ wall is monitored along ROIs (as indicated in Figure 10), leading to a measure of mixing performance. The farther the flow needs to travel to achieve mixing, categorically corresponding to the minimum of the profile, the less effective the platform. Thus, at 10 μL/min, the analysis show that FDM is the most effective taking approximately 62% of the distance required for the SLA and Polyjet mixers (Figure 11). The blue intensity was measured using the Matlab environment, dividing each ROI (Figure 10) into 8 rectangular equal regions along the width of the channel. For each ROI the first region on the left has been considered and the blue intensity has been measured. The results are shown in Figure 11. In this case the blue intensity can be used as an indicator of mixing for each single section.

Achieving complete mixing over a short period of time is one of main challenges of micro-mixers in microfluidic devices. Increasing the flow rates resulted in longer channel lengths to achieve complete mixing. For very high flow rates of 400 μL/min (Re = 14.81) complete mixing occurred in all three mixers, putting in evidence that a higher mixing efficiency is reached even though the flow is laminar in theory. The Y-shape serpentine microchannel used in this study has smaller dimensions compared to simple Y-shaped microchannels [18] and provides the possibility of testing these high flow rates and channel lengths, which is a main advantage of 3D printed fluidics compared with classical soft lithography devices relying of planar photolithographic templates.

Table 2 presents a comparison of the three types of micro-mixers. It is interesting to compare the results included in the present paper to the results shown in [18]. The channel size is 0.5 in [18] mm and 0.6 in the present paper. The flow rates tested in [18] are 25, 50 and 100 μL/min while in the present paper 10, 50, 200 and 400 μL/min are tested. Almost in both, the experimental setups FDM requires the shortest channel length, maybe due to roughness of the FDM channel, and so making an apparent limitation to the 3D printing technology becoming beneficial. The presence of macroscopic roughness, due to the physic of the process itself, on the FDM channel surface can act as ridges and cause stretching and folding of the flow as described in [29]. Ridges can cause chaotic advection which leads to increased mixing, thus reducing the required channel length to achieve complete mixing [25]. However, some important differences must be highlighted: (1) in the experiments reported in the present paper, for higher flow rates the best performance is obtained by SLA, with the same result as the low flow rate, while FDM and polyjet had a decrease of the performance; (2) for intermediate flow rates of 50 and 100 μL/min no complete mixing occurred at all. This last phenomenon is not described in [18] and can be explained with a diffusion time, for complete mixing in laminar flow, higher than the time between inlet and outlet. According to diffusion and laminar flow theories complete mixing should occur at a length respectively 5 times and 20 times higher than in the 10 μL/min case, then agreeing with the lack of mixing. At 400 μL/min a new phenomenon rather than diffusion occurs, but it cannot be turbulent flow due to the low Reynolds number, equal to 14.81, shown in Table 2. Probably, this is due to chaotic advection but further studies must be carried on to understand this behaviour.

## 4. Conclusions

This research leads us to conclude that SLA, FDM and polyjet processes are suitable for microfluidic mixing devices. The complete mixing is achieved for all of them in two distinct situations: for Re equal to 0.37 and equal to 15, which should still represent a laminar flow. As a consequence, diffusive mixing cannot justify the experimental behaviour. In fact, while diffusion is clearly the most important mixing phenomenon up to Re equal to 7.4, between 7.4 and 14.8 the behaviour changes, probably due to the chaotic advection phenomenon. In this context, FDM and polyjet behaved better at low flow rate than higher ones, while SLA had the same behaviour for lower and higher flowrates. Further studies on the relationship between surface micro and macro geometry, flow speed and completeness of mixing must be conducted through new tests with different flow rates and new channel micro and macro geometries. 

## Figures and Tables

**Table 1:** Process parameters and materials used for the fabrication of the micro-mixers.

Printing Method	Layer Height (mm)	Laser Spot Size or Nozzle Diameter or Resolution	Filling or Etching Strategy	Type of Material
Polyjet	0.028	600 DPI ( *x* and *y*-axes) 900 DPI (*z*-axis)	solid, glossy finishing	Vero White Plus UV Photopolymeric Resin
SLA, Stereolithography	0.025	0.14 (mm)	solid	Clear Form V2 UV Photopolymer Resin
FDM, Fused deposition modelling	0.090	0.40 (mm)	raster	Fabbrix natural PLA

**Table 2:** Required length for each Y-shape serpentine micro-mixer fabricated by three methods where a relative intensity was reached, which can be considered complete mixing.

Methods	Flow Rate = 10 (μL/min) Re = 0.37 Length ^1^ (mm or Stage)	Flow Rate = 50 (μL/min) Re = 1.85 Length (mm or Stage)	Flow Rate = 200 (μL/min) Re = 7.4 Length (mm or Stage)	Flow Rate = 400 (μL/min) Re = 14.81 Length (mm or Stage)
Polyjet	150.6 (ROI 8)	-	-	197.6 (ROI 10)
SLA	150.6 (ROI 8)	-	-	150.6 (ROI 8)
FDM	80.6 (ROI 5)	-	-	197.6 (ROI 10)

^1^ Obtained values in mm calculated for different regions of interest (ROIs).
